# Supplementary material for: Empirical methods for controlling false positives and estimating confidence in ChIP-Seq peaks
Source: BMC Bioinformatics. 2008 Dec 5;9:523. doi: 10.1186/1471-2105-9-523 (PMC2628906; doi:10.1186/1471-2105-9-523)
Supplement: Additional File 1 — A variety of html documents from the USeq web site detailing the available applications, their best usage, output file type descriptions, command line menus, etc. [file 1471-2105-9-523-S1.zip › USeqUserGuides/JavaDoc.zip!/JavaDoc/edu/utah/seq/methylation454/StatMethylationData.html]

StatMethylationData


|  |  |  |
| --- | --- | --- |
| **Overview** \| **Package** \| **Class** \| **Use** \| **Tree** \| **Deprecated** \| **Index** \| **Help** | |  |
| **PREV CLASS**   NEXT CLASS | **FRAMES**    **NO FRAMES**     **All Classes** |  |
| SUMMARY: NESTED \| FIELD \| CONSTR \| METHOD | DETAIL: FIELD \| CONSTR \| METHOD |  |


---


## edu.utah.seq.methylation454 Class StatMethylationData

```
java.lang.Object
  edu.utah.seq.methylation454.StatMethylationData
```

---

``` public class StatMethylationData extends java.lang.Object ```

---

**Constructor Summary**

`StatMethylationData(java.lang.String[] args)`


| **Method Summary** | |
| --- | --- |
| `double` | `calculateWilcoxon(float[] one, float[] two)` |
| `static java.util.ArrayList` | `findCommonAmplicons(Sample one, Sample two)` |
| `static java.util.ArrayList` | `findCommonCpGs(Amplicon one, Amplicon two)` |
| `static void` | `main(java.lang.String[] args)` |
| `void` | `statCpGs(Sample one, Sample two)`             Generates two tailed Chi-square p-values for differences in %C (a proxy for %methylation) for each CpG in each Amplicon between two samples and a Wilcoxon Signed Rank p-value for all of the CpG %Cs in an amplicon. |

**Methods inherited from class java.lang.Object**

`equals, getClass, hashCode, notify, notifyAll, toString, wait, wait, wait`

**Constructor Detail**

### StatMethylationData

```
public StatMethylationData(java.lang.String[] args)
```


**Method Detail**

### main

```
public static void main(java.lang.String[] args)
```

---


### statCpGs

```
public void statCpGs(Sample one,
                     Sample two)
```

:   Generates two tailed Chi-square p-values for differences in %C (a proxy for %methylation) for each CpG in each Amplicon between two samples
    and a Wilcoxon Signed Rank p-value for all of the CpG %Cs in an amplicon.

---


### calculateWilcoxon

```
public double calculateWilcoxon(float[] one,
                                float[] two)
```

---


### findCommonCpGs

```
public static java.util.ArrayList findCommonCpGs(Amplicon one,
                                                 Amplicon two)
```

---


### findCommonAmplicons

```
public static java.util.ArrayList findCommonAmplicons(Sample one,
                                                      Sample two)
```


---


|  |  |  |
| --- | --- | --- |
| **Overview** \| **Package** \| **Class** \| **Use** \| **Tree** \| **Deprecated** \| **Index** \| **Help** | |  |
| **PREV CLASS**   NEXT CLASS | **FRAMES**    **NO FRAMES**     **All Classes** |  |
| SUMMARY: NESTED \| FIELD \| CONSTR \| METHOD | DETAIL: FIELD \| CONSTR \| METHOD |  |


---
